# Multi-scanner and multi-modal lumbar vertebral body and intervertebral disc segmentation database

**DOI:** 10.1038/s41597-022-01222-8

**Published:** 2022-03-23

**Authors:** Yasmina Al Khalil, Edoardo A. Becherucci, Jan S. Kirschke, Dimitrios C. Karampinos, Marcel Breeuwer, Thomas Baum, Nico Sollmann

**Affiliations:** 1 Biomedical Engineering Department, Eindhoven University of Technology, Eindhoven, The Netherlands; 2 Department of Diagnostic and Interventional Neuroradiology, School of Medicine, Klinikum rechts der Isar, Technical University of Munich, Munich, Germany; 3 TUM-Neuroimaging Center, Klinikum rechts der Isar, Technical University of Munich, Munich, Germany; 4 Department of Diagnostic and Interventional Radiology, University Hospital Ulm, Ulm, Germany; 5 Department of Diagnostic and Interventional Radiology, School of Medicine, Klinikum rechts der Isar, Technical University of Munich, Munich, Germany; 6 Department of Radiology and Biomedical Imaging, University of California San Francisco, San Francisco, CA USA

**Keywords:** Bone, Diagnostic markers

## Abstract

Magnetic resonance imaging (MRI) is widely utilized for diagnosing and monitoring of spinal disorders. For a number of applications, particularly those related to quantitative MRI, an essential step towards achieving reliable and objective measurements is the segmentation of the examined structures. Performed manually, such process is time-consuming and prone to errors, posing a bottleneck to its clinical applicability. A more efficient analysis would be achieved by automating a segmentation process. However, routine spine MRI acquisitions pose several challenges for achieving robust and accurate segmentations, due to varying MRI acquisition characteristics occurring in data acquired from different sites. Moreover, heterogeneous annotated datasets, collected from multiple scanners with different pulse sequence protocols, are limited. Thus, we present a manually segmented lumbar spine MRI database containing a wide range of data obtained from multiple scanners and pulse sequences, with segmentations of lumbar vertebral bodies and intervertebral discs. The database is intended for the use in developing and testing of automated lumbar spine segmentation algorithms in multi-domain scenarios.

## Abstract (summary)

**Table:** 

Measurement(s)	Vertebral Body • Intervertebral Disc
Technology Type(s)	Magnetic Resonance Imaging

## Background & Summary

Magnetic resonance imaging (MRI) is the modality of choice for detecting and visualizing almost all spinal disorders, as it provides non-invasive soft tissue visualization with excellent contrast, more detailed compared to other modalities^1–5^. Thus, it is widely utilized in orthopedic and neurosurgical diagnostics, ranging from dedicated imaging in scoliosis, intervertebral disc disease, and osteoporosis to injuries including vertebral fractures as well as to computer-assisted surgical intervention planning and guidance^1–4,6^.

Visual image reading represents the standard approach in the clinical setting for evaluation of routine spine MRI. Reporting derived from visual image assessment by the radiologist may be enhanced by using the approach of structured reporting (e.g., use of predefined formats and terms to create radiological reports, using a high level of standardized and organized information in template context^7–9^) and by implementing semi-quantitative grading schemes (e.g., Pfirrmann classification for lumbar disc degeneration^10^). Going one step further, generating quantitative measures for specific anatomical structures along the spine would be welcome to be able to provide meaningful objective markers related to spinal disorders, which could facilitate patient phenotyping, clinical management, and adequate treatment selection. A robust and precise segmentation of vertebral bodies and intervertebral discs is a major step towards a reliable diagnosis of various conditions in automated and computer-assisted systems, as well as for quantitative MRI regarding extraction of image-based markers^11–15^.

However, automated spine segmentation has not yet made the transition to clinical routine and remains a challenging problem due to the variable and complex shape of the spine anatomy, as well as the presence of noise and other artefacts in imaging data^6,16^. Moreover, routine spine MRI acquisitions pose several challenges for achieving robust and accurate segmentations, which is mostly due to some unavoidable MRI acquisition characteristics and pitfalls. Specifically, these include the presence of partial volume effects related to anisotropic spatial resolution, non-homogeneous intensities between central and marginal areas of the spine due to bias field artefacts, and the non-existence of standardized measurement units (unlike Hounsfield Units as used for computed tomography [CT])^6^. This is additionally highlighted in large multi-site studies, where variations in scan parameters often produce images that vary significantly in contrast and quality, especially in cases where multiple diagnostic MRI sequences are used among different clinical practices^17,18^.

Up to now, diagnostic and analysis methods of MRI data predominantly rely on manual annotation of anatomical objects, such as vertebrae and intervertebral discs. This process is time-consuming and subjective, and often prone to intra- and inter-annotator variability^17,19^. Of note, the high time expenditure of manual segmentation may hamper direct use, particularly when multi-level segmentations are desired (e.g., segmentation of the entire lumbar spine). Thus, automating the segmentation process would strongly benefit clinicians and scientists, especially for large-scale and multi-site studies targeting quantitative MRI. However, a reliable automated segmentation method, which has the potential to be included in standard clinical routines in the future, has to be able to generalize to the large variety of MRI sequences and parameter settings, while being reasonably fast and not overly complex to operate. This remains a difficult challenge, despite the surge in recent developments of automated segmentation techniques^18,20^.

Most existing methods focus on one particular pulse sequence, which is not sufficient to adequately acknowledge the frequent multi-sequence acquisitions in clinical routine, or they may suffer from considerably long computational time, as well as the requirement for user input and navigation^21,22^. Other methods require extensive prior knowledge, such as shape information, to construct a representative preliminary model of anatomical shape from training data and to approximate optimization to new data^16,23,24^. Such methods are clearly limited by prior knowledge, which – in the case of large-scale and multi-site studies – should contain enough representative information to handle all possible variations in data. Current constraints in data collection and data sharing pose a challenge in acquiring enough of such data, thus producing highly specific models that are often only applicable to a small variety of cases. Automated medical image analysis algorithms should be robust enough to inherent data variability to ensure their successful integration into existing infrastructure, with the long-term perspective of becoming clinically feasible. However, without enough representative data comprising all of the diverse variations in spine MRI, algorithm generalizability is hard to achieve.

Recent advances in deep learning-based segmentation methods hold a lot of promise to alleviate the challenges described above^21,25–28^. This seems especially true for improving the generalization on multi-site and multi-scanner data. However, used algorithms require large datasets annotated by experts for both development and testing. Yet, publicly available datasets of annotated vertebrae and intervertebral discs, collected from multiple scanners and different acquisition protocols, are very limited. Therefore, the purpose of this article and its related dataset is to provide a reference database containing a wide range of MRI data obtained from multiple scanners and involving various pulse sequences, along with the segmentations of lumbar vertebral bodies (L1 to L5) and intervertebral discs.

Using a database like the herein presented, automatization in image analysis and processing could be further facilitated, which could directly influence the field of radiology and pave the way towards semi-automated and fully-automated algorithms for radiological diagnostics. As such, over the recent years, automated spine image analysis has seen a growing interest, particularly for the detection of vertebral fractures^29^, assessment of spinal deformities^21^, and computer-assisted surgical interventions^30^. As the lumbar spine is a particularly common site for various spinal disorders that can cause chronic low back pain (LBP), segmentation of anatomical structures along the lumbar spine is of considerable interest. While many factors can contribute to spinal disease and deformation, such as fractures, accidental injuries, osteoporosis, vertebral neoplasm or scoliosis^31^, the primary cause of chronic LBP in most patients is believed to be related to spinal degeneration, particularly of lumbar intervertebral discs and endplates^32–37^. Spinal degeneration is a natural consequence of aging, but it can be accelerated by trauma, repetitive stress, systemic disease, and other factors^35,38–41^. Thus, investigating the relevance between chronic LBP pathology and lumbar vertebrae and intervertebral disc morphology and composition using specific segmentations could aid in developing appropriate methods to assist early diagnosis, provide better surgical planning, and facilitate individualized treatment strategies and patient phenotyping.

In summary, we offer a database of manually segmented lumbar vertebral bodies and intervertebral discs in MRI datasets collected from a variety of scanners and using different pulse sequence protocols. Besides the images that are part of standard scanning routines, such as non-contrast-enhanced and contrast-enhanced T1-weighted, T2-weighted, and short tau inversion recovery (STIR) images, we provide the access to images obtained with a DIXON turbo spin-echo (TSE) sequence. Thus, combined with other available imaging sequences, this data could help in the development of more efficient and robust methods of segmenting musculoskeletal structures, and it could facilitate quantitative MRI by achieving automated segmentation and analysis of datasets, which is useful for assessing vertebral bodies (e.g., fat fraction^42–46^), cartilage endplates (e.g., T2* ^47,48^), and intervertebral discs (e.g., T1rho mapping^44,48,49^). The data including segmentations can be used as training and test datasets for (semi-)automated algorithms, which can potentially benefit from the heterogeneous character of this study’s database, as it fosters the development of approaches that are more generalizable to data derived from multiple sites and scanners, which is known to be a major challenge for models that are typically trained on highly homogeneous data.

## Methods

### Patient cohort

This retrospective study was approved by the local Institutional Review Board (Ethikkommission der Technischen Universität München). The requirement for written informed study consent was waived due to the retrospective character of the specific analyses used for this publication. Yet, all patients provided standard informed consent for MRI scanning during clinical routine and agreed on the day of visit for MRI acquisition on an opt-in basis that their data may be used for scientific purposes. The patients were informed that their data may be anonymously publicly shared without any personal identifiable information, and the analyses for this study were performed on de-identified data.

Thirty-four patients (mean age: 60.4 ± 15.2 years, age range: 30.0–88.1 years, 58.8% females) with the following medical indications for MRI acquisition during the clinical routine were included in this study: 1) LBP with or without radiculopathy due to suspected spinal degeneration (41.3% of patients), 2) postoperative or follow-up imaging after resection of a spinal tumor (23.5% of patients), 3) known malignancy with (suspected) spinal metastases (17.6% of patients), 4) spondylodiscitis or other spinal inflammatory/infectious diseases (14.7% of patients), and 5) spinal fracture (2.9% of patients). Patient characteristics (sex and age) and medical indications are listed in Table 1.

**Table 1:** Patient characteristics with clinical indications.

ID	Gender	Age	Clinical indication	Clinical indication code*
02	Female	51.1	FU resection ependymoma	1
04	Male	38.3	Postop resection neurinoma	1
05	Female	30.0	FU resection ependymoma	1
07	Male	77.2	LBP and radiculopathy	2
10	Female	73.6	FU resection neurinoma	1
11	Female	40.1	Mamma-Ca with spinal metastases	3
12	Male	48.1	FU resection ependymoma	1
13	Female	52.3	Screening spinal tumor	3
14	Male	57.2	FU inflammatory lesion	4
15	Female	82.4	LBP	2
16	Male	61.2	Thymus-Ca with spinal metastases	3
17	Female	70.0	Spondylodiscitis	4
18	Female	61.1	LBP and radiculopathy	2
19	Female	71.7	Lung-Ca with spinal metastases	3
20	Male	48.9	Radiculopathy S1 left	2
21	Male	71.0	Radiculopathy L4 (both sides)	2
22	Female	86.5	FU resection meningioma	1
23	Male	45.9	Radiculopathy S1 right	2
24	Female	72.1	Sacral fracture	5
25	Female	52.9	Lung-Ca with spinal metastases	3
26	Female	40.9	LBP and radiculopathy	2
27	Female	49.1	LBP and radiculopathy	2
28	Male	64.0	Spondylodiscitis	4
29	Female	88.1	Mamma-Ca with spinal metastases	3
30	Male	81.7	LBP and radiculopathy	2
31	Male	55.7	LBP and radiculopathy	2
32	Male	39.8	Postop resection meningioma	1
33	Male	62.3	LBP and radiculopathy	2
34	Female	57.2	Radiculopathy L5 left	2
35	Male	69.6	Spondylodiscitis	4
36	Female	56.0	FU resection ependymoma	1
37	Female	79.1	LBP and radiculopathy	2
38	Female	75.8	LBP and radiculopathy	2
39	Female	43.1	Spondylodiscitis	4

*Clinical indication code legend: 1 Postoperative/follow-up (FU) imaging for tumor after resection, 2 Low back pain (LBP) with or w/o radiculopathy due to degenerative changes, 3 Malignancy with (suspected) spinal metastases, 4 Spondylodiscitis or other inflammation/infection, 5 Trauma/fracture.

The database contains MRI datasets collected from these 34 patients, whereby 21 patients were rescanned at a second time point using a different MRI scanner model and/or sequence protocol (mean interval between first and second MRI scan: 8.3 ± 12.6 months, interval range: 0.5–60.8 months). From the remaining 13 patients only MRI datasets of one imaging time point were used. MRI scans acquired between August 2014 and October 2019 were considered in this study, with the data being acquired either on institutional-intern scanners or on scanners of other institutions, thus being available after image transfer due to clinical requests. A detailed list of pulse sequences and MRI vendors per patient, together with the time intervals between scans, are shown in Tables 2 and 3.

**Table 2:** Scan characteristics with image sequences per scanner vendor and model type for the first scan.

ID	No. of scans	MRI 1 vendor/model	MRI 1 sequence*
02	1	Philips Achieva	T1-w, nc T1-w, T2-w Dix.
04	2	Siemens Espree	T1-w, nc T1-w, STIR
05	2	Philips Achieva	T1-w, nc T1-w
07	2	Siemens Avanto	nc T1-w, T2-w, STIR
10	2	Siemens Verio	nc T1-w, T1-fs, T2-w
11	1	Philips Achieva	T1-w, nc T1-w, T2-w Dix.
12	2	Philips Ingenia	T1-w, nc T1-w
13	1	Philips Achieva	T1-w, nc T1-w, T2-w Dix.
14	1	Philips Achieva	T1-w, nc T1-w, T2-w Dix.
15	2	Siemens Symphony	nc T1-w, T2-w
16	2	Siemens Avanto	nc T1-w, T2-w, STIR
17	1	Philips Achieva	T1-w, nc T1-w, T2-w Dix.
18	1	Philips Achieva	nc T1-w, T2-w Dix.
19	2	Siemens Avanto	nc T1-w, T2-w
20	1	Philips Achieva	nc T1-w, T2-w Dix.
21	2	Siemens Amira	nc T1-w, T2-w
22	2	Siemens Verio	T1-w, nc T1-w, T2-w
23	1	Philips Achieva	T1-w, nc T1-w, T2-w Dix.
24	1	Philips Elition	nc T1-w, T2-w Dix.
25	1	Philips Achieva	T1-w, nc T1-w, T2-w Dix.
26	1	Philips Achieva	nc T1-w, T2-w Dix.
27	1	Philips Achieva	T1-w, nc T1-w, T2-w Dix.
28	2	Siemens Verio	nc T1-w, T2-w, STIR
29	2	Philips Achieva	T1-w, nc T1-w, T2-w Dix.
30	2	Siemens Espree	nc T1-w, T2-w
31	1	Philips Ingenia	nc T1-w, T2-w Dix.
32	2	Philips Elition	T1-w, nc T1-w, T2-w Dix.
33	2	Siemens Aera	nc T1-w, T2-w
34	2	Philips Achieva	nc T1-w, T2-w Dix.
35	2	Philips Elition	nc T1-w
36	2	Siemens Magnetom	T1-w, nc T1-w, T2-w
37	2	Philips Achieva	nc T1-w, T2-w
38	2	GE Signa	nc T1-w, T2-w
39	2	Siemens Avanto	T1-w, nc T1-w, T2-w, STIR

*T1-w, nc T1-w, T2-w, T1-fs, STIR, and T2-w Dix. stand for T1-weighted contrast-enhanced, T1-weighted non-contrast-enhanced, T2-weighted, T1-weighted fat-saturated, short tau inversion recovery, and T2-weighted Dixon sequences.

**Table 3:** Scan characteristics with image sequences per scanner vendor and model type for the follow-up scan.

ID	T*	MRI 2 vendor/model	MRI 2 sequence**
04	2.8	Philips Ingenia	T1-w, nc T1-w, T2-w Dix.
05	15.6	Siemens Verio	T1-w, nc T1-w, T2-w, STIR
07	13.7	Philips Achieva	nc T1-w, T2-w Dix.
10	4.5	Philips Ingenia	T1-w, nc T1-w, T2-w Dix.
12	9.3	Philips Achieva	T1-w, nc T1-w, T2-w Dix.
15	1.6	Philips Achieva	T1-w, nc T1-w, T2-w Dix.
16	0.7	Philips Achieva	T1-w, nc T1-w, T2-w Dix.
19	2.8	Philips Achieva	T1-w, nc T1-w, T2-w Dix.
21	6.1	Philips Elition	nc T1-w, T2-w Dix.
22	8.7	Philips Elition	nc T1-w, T2-w Dix.
28	0.5	Philips Achieva	nc T1-w, T2-w Dix.
29	6.2	Philips Ingenia	T1-w, nc T1-w, T2-w Dix.
30	12.2	Philips Achieva	T1-w, nc T1-w, T2-w Dix.
32	0.7	Philips Achieva	T1-w, nc T1-w, T2-w Dix.
33	13.7	Philips Achieva	nc T1-w, T2-w Dix.
34	7.1	Siemens Avanto	nc T1-w, T2-w, STIR
35	2.7	Philips Ingenia	T1-w, nc T1-w, T2-w Dix.
36	2.8	Philips Achieva	nc T1-w, T2-w Dix.
37	1.6	Philips Achieva	nc T1-w, T2-w Dix.
38	60.8	Philips Achieva	nc T1-w, T2-w Dix.
39	1.1	Philips Achieva	T1-w, nc T1-w, T2-w Dix.

*T is the time interval between the two scans. **T1-w, nc T1-w, T2-w, T1-fs, STIR, and T2-w Dix. stand for T1-weighted contrast-enhanced, T1-weighted non-contrast-enhanced, T2-weighted, T1-weighted fat-saturated, short tau inversion recovery, and T2-weighted Dixon sequences.

### Magnetic resonance imaging

Various scanner models from different vendors were utilized for collecting a number of images, including non-contrast-enhanced T1-weighted, contrast-enhanced T1-weighted, T2-weighted, STIR, T2-weighted DIXON, and T1-weighted fat-saturated (fs) images. 70.9% of included datasets were derived from Philips scanners (Achieva, Ingenia, and Elition), 27.3% were derived from Siemens scanners (Avanto, Verio, Espree, Symphony, Amira, Aera, and Magnetom). One dataset (1.8%) was taken from a GE scanner (Signa).

The field of view (FOV) covered at least the lumbar spine. Only scans that were performed in supine position were included. Each patient had at least one MRI scan performed on a Philips system with the following reference sequences:

- Sagittal non-contrast-enhanced T1-weighted sequence: repetition time (TR)/echo time (TE) = 600/8 ms, FOV = 180 × 275 × 49 mm, acquisition voxel size = 0.80 × 1.00 × 3.00 mm^3^, acquisition duration = 3 min 3 s.
- Sagittal T2-weighted DIXON TSE sequence: TR/TE = 2,500/100 ms, FOV = 180 × 275 × 49 mm, acquisition voxel size = 0.70 × 0.98 × 3.00 mm^3^, acquisition duration = 3 min 25 s.

### Image segmentation

Non-contrast-enhanced T1-weighted images were used for obtaining the segmentations of lumbar vertebral bodies (L1 to L5) and intervertebral discs (L1/2 to L4/5) for each patient and each respective MRI dataset. Segmentations were performed manually and without (semi-)automatic software support. Specifically, non-contrast-enhanced T1-weighted images were successively opened in MITK, an open-access image viewer software package that allows a simultaneous visualization of the images in all three image planes ([http://mitk.org/wiki/The_Medical_Imaging_Interaction_Toolkit_(MITK)]; German Cancer Research Center, Division of Medical and Biological Informatics, Medical Imaging Interaction Toolkit, Heidelberg, Germany). The manual segmentations were done in the sagittal plane for each vertebral body and intervertebral disc, respectively. Segmentation was performed by a medical doctor. Regions of interest (ROIs) were carefully drawn at the boundaries of the vertebrae and intervertebral discs, respectively. The other image planes were used to check for accidentally included structures not belonging to the vertebral bodies or intervertebral discs. In these cases, ROIs were corrected accordingly. For lumbar vertebral bodies, each vertebrae was separately enclosed in sagittal slices, avoiding any inclusion of paraspinal tissue or fluid. Posterior elements were not considered, thus restricting the segmentations to the vertebral corpora only. Analogously, lumbar intervertebral discs were also separately segmented in sagittal slices, considering the entire disc of the whole circumference. All segmentations were supervised by a radiologist with eleven years of experience. Segmentation time per one T1-weighted image series amounted to 60–90 min.

The obtained segmentation labels, generated in non-contrast-enhanced T1-weighted images of all patients of one or two MRI sessions, were then overlaid over the rest of the available sequences acquired with different MRI scanners and pulse protocols. Due to the differences in imaging and scanning parameters, the original images were registered to the non-contrast-enhanced T1-weighted images using the 3D linear registration tool in ITK-SNAP (https://www.itksnap.org), ensuring that labels were accurately overlaid. We resampled each scan in the space of the respective non-contrast-enhanced T1-weighted image using linear interpolation with an identity transform^50,51^, given that we have the case of two or more scans of the same patient with different coordinate transformations to patient space. Once resampled, additional verification was performed to ensure all segmentation masks are accurately overlaid over all available sequences per patient. The verified data were subsequently uploaded to the database.

**Fig. 1:**
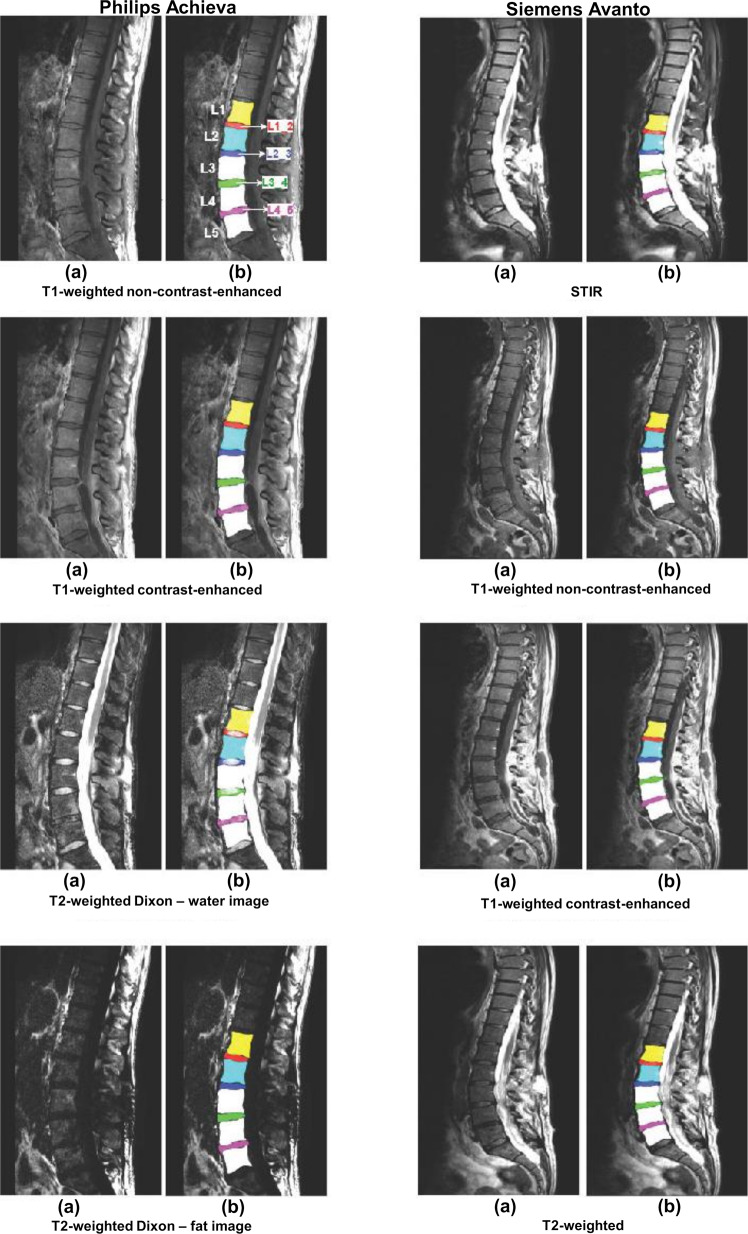
Segmented lumbar vertebral bodies (L1 to L5) and intervertebral discs (L1_2, L2_3, L3_4, and L4_5) per sequence. One middle slice of each sequence is shown in (**a**) with corresponding segmentation masks in (**b**).

**Fig. 2:**
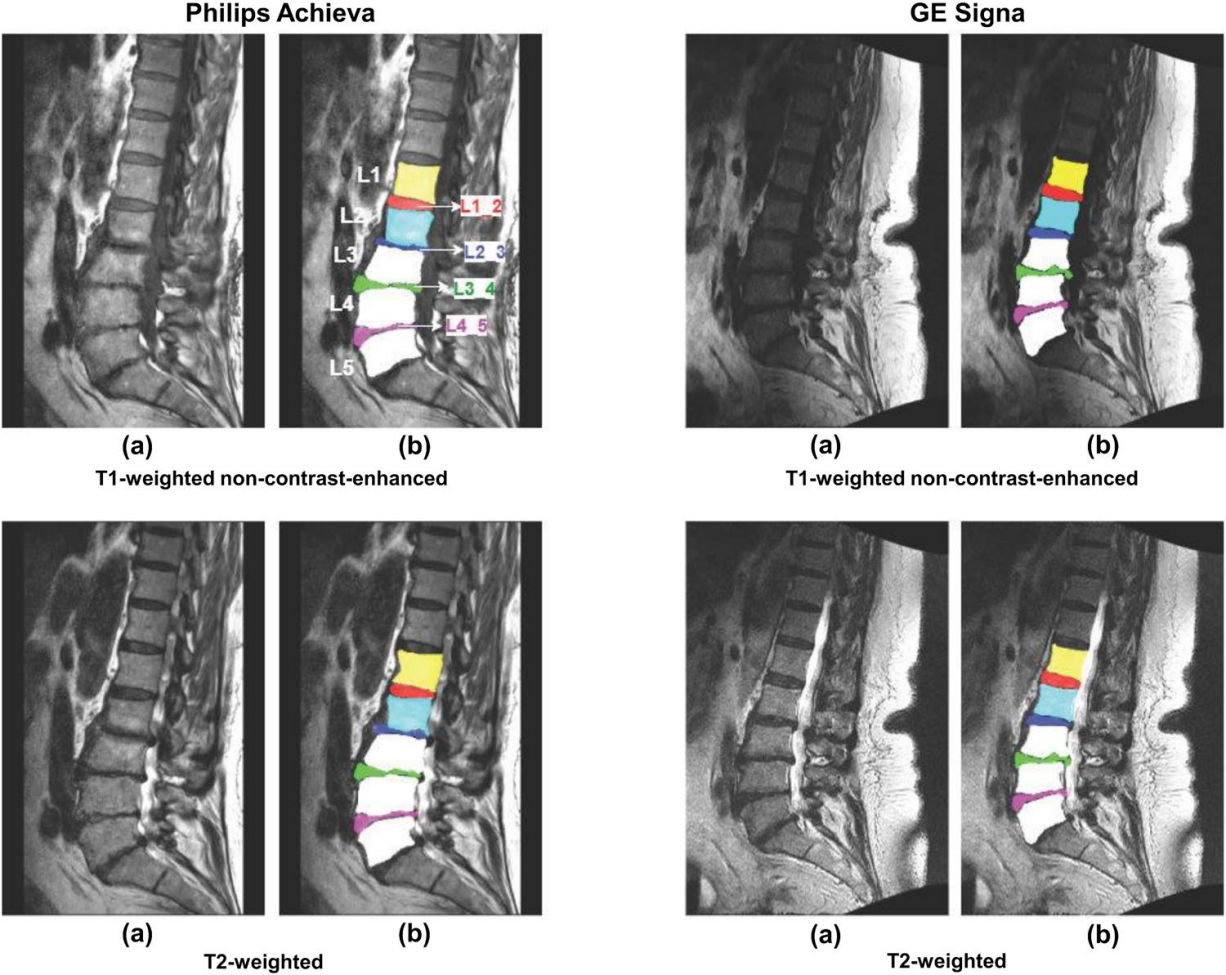
Segmented lumbar vertebral bodies (L1 to L5) and intervertebral discs (L1_2, L2_3, L3_4, and L4_5) per sequence acquired from two different scanner vendors. Significant qualitative differences arise due to scanner and protocol variation. One middle slice of each sequence is shown in (**a**) with corresponding segmentation masks in (**b**).

**Fig. 3:**
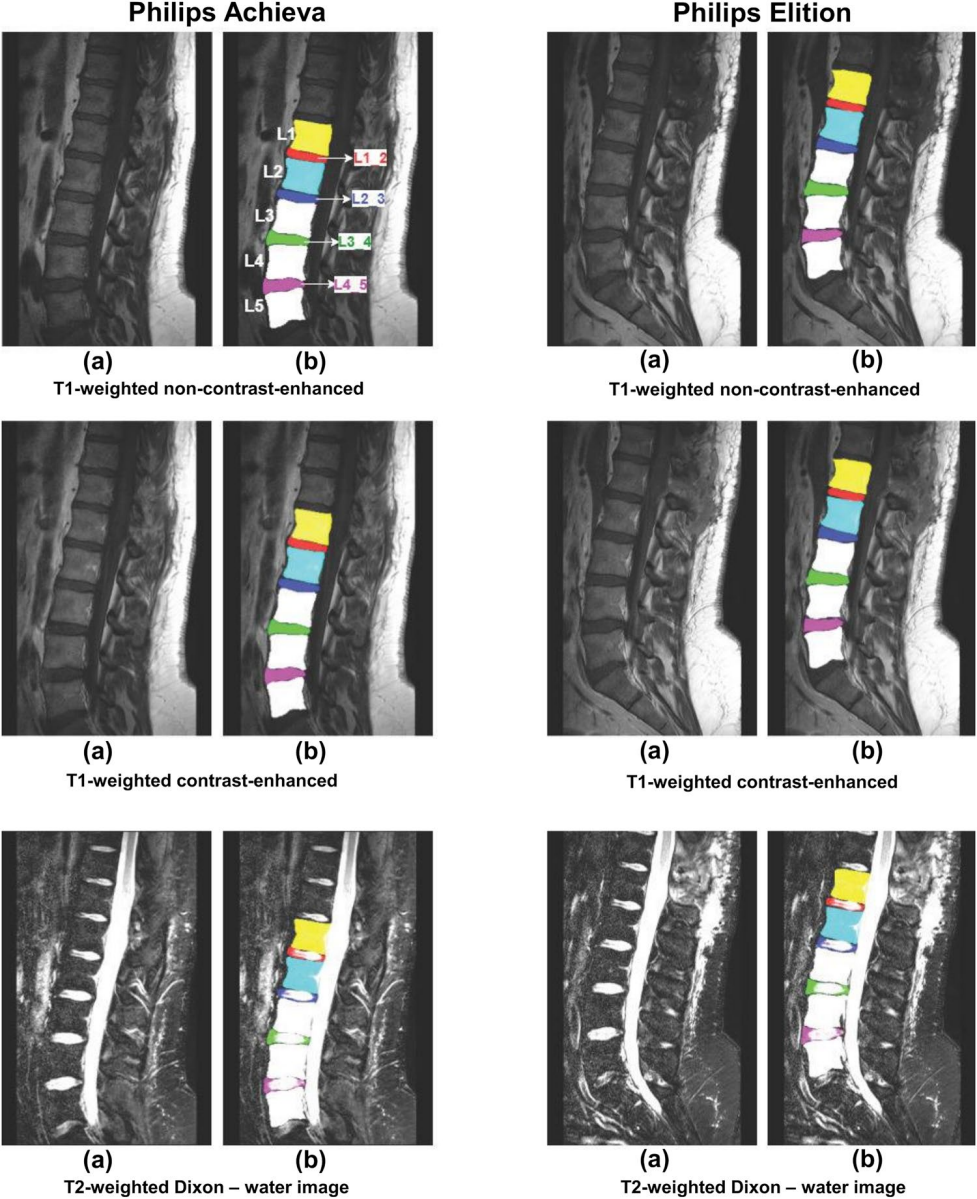
Segmented lumbar vertebral bodies (L1 to L5) and intervertebral discs (L1_2, L2_3, L3_4, and L4_5) per sequence acquired from two different scanner models belonging to the same vendor. One middle slice of each sequence is shown in (**a**) with corresponding segmentation masks in (**b**).

**Fig. 4:**
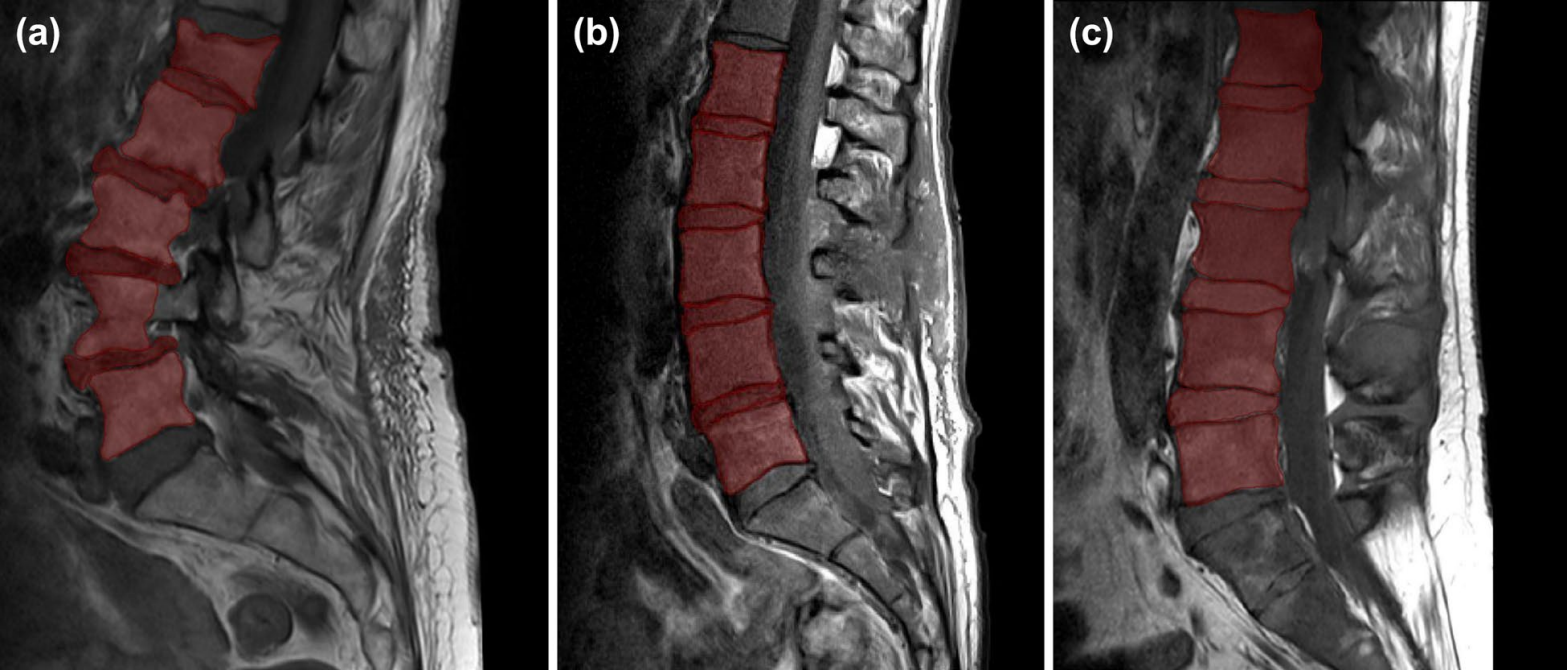
Segmented lumbar vertebral bodies (L1 to L5) and intervertebral discs (L1_2, L2_3, L3_4, and L4_5) using sagittal T1-weighted sequences in a patient with a fractured vertebral body L1 (**a**), a patient with spondylodiscitis of the segment L4/L5 (**b**), and a patient with diffuse metastatic lesions in vertebral bodies (**c**). Individual segmentation masks for single vertebral bodies and intervertebral discs are outlined in red.

Examples of segmented lumbar vertebral bodies and intervertebral discs are shown in Figs. 1–4. In detail, Fig. 1 shows two scans acquired on scanners from differing vendors (Philips and Siemens) per each available sequence. Similar is shown in Fig. 2 for another vendor (Philips and GE). Furthermore, Fig. 3 shows the differences between scans of the same patient acquired from the same vendor’s scanners (Philips), which differ in models. Figure 4 provides three exemplary patient cases with segmentations shown on T1-weighted imaging in presence of pathological findings that may render manual segmentations particularly demanding (e.g., vertebral fracture, spondylodiscitis, and metastatic bone lesions).

The manual segmentations of each vertebral body and intervertebral disc are available as separate binary masks, where pixels with an intensity value of 1 correspond to the tissue of interest, while pixels of an intensity value of 0 belong to the background. Each mask of each image volume was stored as a separate *.nii file. In total, each available volume is accompanied with the corresponding segmentations of lumbar vertebral bodies and intervertebral discs.

## Data Records

The database is available online within the OSF Repository, including the different imaging datasets, segmentation files, as well as metadata comprising patient characteristics and details on the available pulse sequences and used MRI systems per patient (Identifier: 10.17605/OSF.IO/QX5RT)^52^. Furthermore, Python scripts to read and visualize the data by utilizing open-source image analysis libraries – SimpleITK (https://simpleitk.org/) and NiBabel (https://nipy.org/nibabel/) – are provided.

Non-contrast-enhanced and contrast-enhanced T1-weighted, T2-weighted, STIR, and T1-weighted fs images are stored as separate datasets for each patient per scanner model. In the case of the T2-weighted DIXON sequences, sagittal water, fat, and in-phase images are deposited as separate datasets for each patient, if available. The segmentation maps of each vertebra (L1 to L5) and intervertebral discs (L1/2 to L4/5) are stored as *.nii files. All imaging data, as well as the segmentation maps, are saved using the Neuroimaging Informatics Technology Initiative (NIfTI) format (https://nifti.nimh.nih.gov/). NIfTI files have several features, such as raw data saved in 3D, containing two affine coordinates to relate voxel to spatial index, as well as additional data such as key acquisition parameters, encoding directions, and grid spacing, saved as a part of the header. NIfTI files can be directly read in a number of programming environments, such as Python, Matlab, and R, as well as directly visualized using tools such as ImageJ (https://imagej.nih.gov/ij/), ITK-SNAP (http://www.itksnap.org/), FSL (https://fsl.fmrib.ox.ac.uk/fsl/fslwiki), AFNI (https://afni.nimh.nih.gov/), and FreeSurfer (https://surfer.nmr.mgh.harvard.edu/).

Datasets of patients and corresponding segmentation masks are labeled with the same patient ID. Masks of each vertebra are labeled as L1 to L5, while the masks for each intervertebral disc are labeled as L1_2, L2_3, L3_4, and L4_5.

## Technical Validation

Acquisition of MRI was medically indicated and image quality control was performed during and/or immediately after completion of the exam by the technologist and radiologist in charge. Furthermore, inclusion of image data in the current study was performed by a board-certified radiologist with eleven years of experience based on a sufficient image quality.

## Data Availability

The database is available online within the OSF Repository, including the different imaging datasets, segmentation files, as well as metadata comprising patient characteristics and details on the available pulse sequences and used MRI systems per patient (Identifier: 10.17605/OSF.IO/QX5RT)^52^. We additionally provide Python scripts to read and visualize the data by utilizing open-source image analysis libraries – SimpleITK (https://simpleitk.org/) and NiBabel (https://nipy.org/nibabel/), available within the repository wiki page.
